# Dataset on water quality characteristics of a hill stream in Bhaderwah, Jammu and Kashmir

**DOI:** 10.1016/j.dib.2019.104462

**Published:** 2019-08-31

**Authors:** Ravinder Kumar, Anil Raina, Neeraj Sharma

**Affiliations:** a Department of Environmental Sciences, University of Jammu, Jammu, 180006, Jammu and Kashmir, India; b Institute of Mountain Environment, University of Jammu, Bhaderwah, 182222, Jammu and Kashmir, India

**Keywords:** Neeru stream, Canadian water quality index, Physicochemical parameters, Heavy metals, Pollution load, Water chemistry, Ion chromatography

## Abstract

The article summarises the data on water quality characteristics of Neeru stream analysed monthly for two years on a seasonal basis averaged to one year. Twenty-five water samples were collected and analysed to understand the quality of water based on index parameters. The data indicates marked variations in the concentration of most constituents mostly in the urban and suburban sections of the stream. The values for Canadian Water Quality Index (CWQI) were within acceptable range except for turbidity and nickel. The tributaries T-1, T-3 (III) and T-4 flowing close to urban settlement revealed relatively high levels of pollution (WQI:85-90), while T-3 (II) bisecting Bhaderwah town was heavily polluted (WQI:82.97). The main water channel (MC-1 to MC-10) with moderate to heavy pollution load in the middle and lower sections revealed reasonably good water quality (WQI 90-95).

**Table:** Specification table

Subject Area	Aquatic Ecology
More Specific subject area	Water quality
Type of data	Tables, figures
How data was acquired	The water samples were collected and analysed following standard protocols and procedures. We used probe-based multi-parameter water analyser, double beam spectrophotometer, Ion Chromatography and volumetric methods for the analysis.
Data form	Raw and analysed
Experimental features	Water quality assessment for different parameters: physical (temperature, discharge, turbidity), chemical (pH, EC, TDS, DO, HCO_3_, F ^–^, Cl ^–^, NO_3_^–^, PO_4_^3 –^, SO_4_^2 –^, Na^+^, NH_4_^+^, K^+^, Ca^2+^, Mg^2+^) and heavy metals (Cu, Ni, Zn Co, Mn) including the WQI in Neeru stream and its tributaries
Data source location	Neeru stream, Bhaderwah, district Doda of Jammu and Kashmir, India
Data accessibility	Whole data set is represented in this communication

**Table:** 

**Value of data** • This data presents a clear view of the water quality characteristics of a hill stream in the pristine, rural and urbanised sections. • The dataset can act as a tool to identify and understand the process and mechanisms affecting surface water chemistry. • The Water Quality Index can be helpful in the assessment and management of water quality. • The database prompts other researchers to take up similar investigations in the adjoining hill streams to have a broader overview of the situation. Real-time monitoring stations can be set up along the urban sections.

## 1 Data

**Fig. 1:**
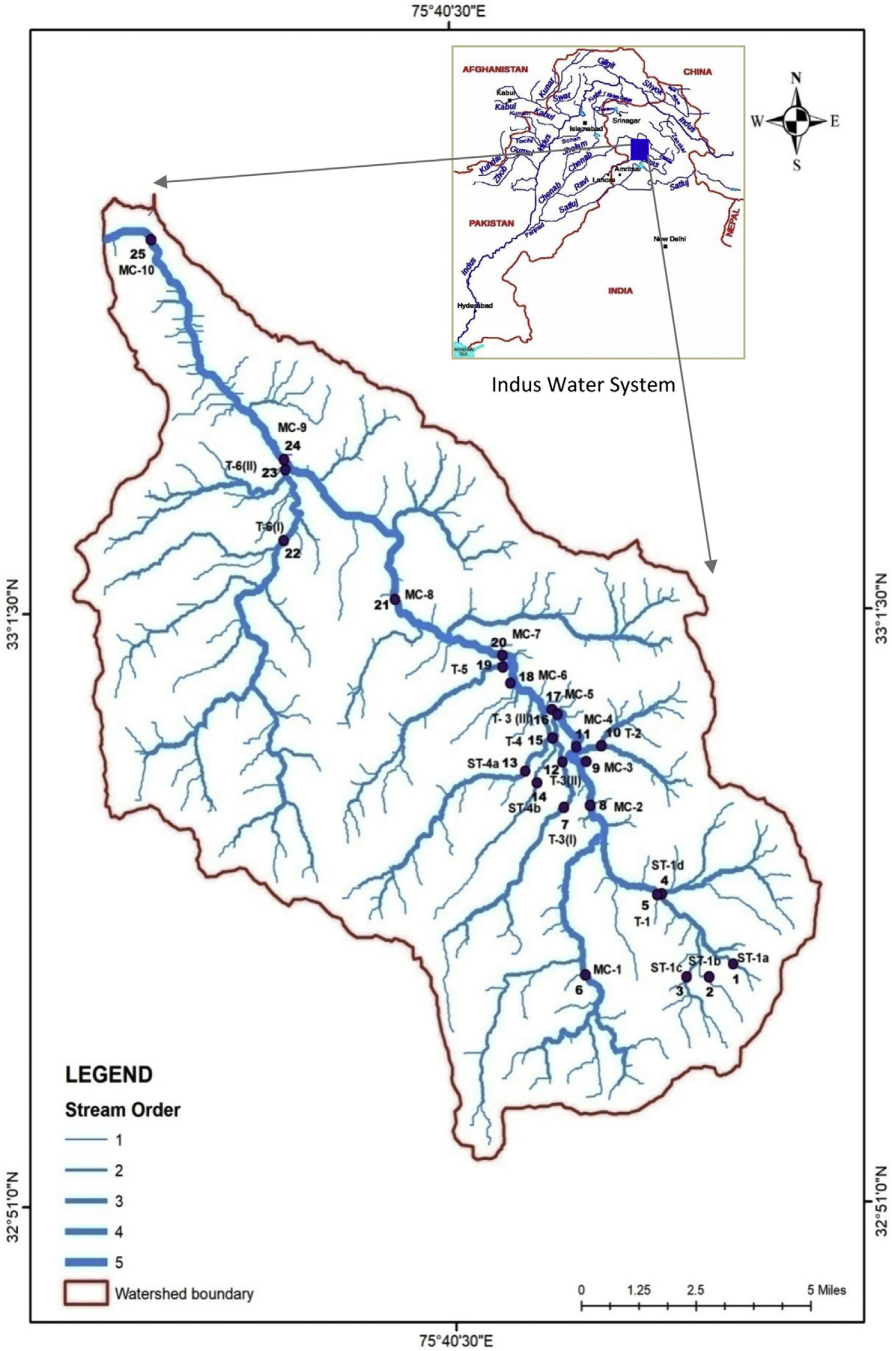
Map showing location of sampling stations along the main channel and tributaries.

The dataset contains 7 Tables and 2 Figs. that represent the quality of stream water for drinking purposes. Table 1 depicts the details of the sampling points interpolated on a map (Fig. 1) with impact stations shown as clusters around the sub-urban and urban centres. The seasonal variations in physical (air & water temperature, discharge and turbidity) and chemical parameters (pH, electrical conductivity, total dissolved solids, dissolved oxygen and bicarbonates) are presented in Table 2 and Table 3 respectively. Observed annual air and water temperature of 25 stations averaged between 4.1°C–26.1°C and 2.7°C–20.5°C, respectively. Relatively high water discharge (729 cubic feet/sec.) was recorded during the spring season, whereas turbidity showed considerable fluctuations (0-61.4 NTU) during the winter (Table 2). pH ranged from neutral to slightly alkaline (7.4-7.9) while conductivity varied between 33.6 μS/cm (summer) and 184.6 μS/cm (winter) and total dissolved solids between 22.1 mg/l (summer) to 137.6 mg/l (autumn). The dissolved oxygen ranged from 7.46 mg/l (summer) to 11.6 mg/l (winter) whereas bicarbonates varied from 10.8 mg/l (summer) to 39.8 mg/l (spring) (Table 3). Among the anions, fluoride varied from below detection limits during winters to 0.46 mg/l in spring while chloride, the important water quality parameter fluctuated between 0.24 mg/l to 9.33 mg/l during spring and winter, respectively. Nitrates ranged between 0 mg/l (autumn) -7.07 mg/l (winter) and sulphate from 0.69 mg/l to 8.86 mg/l during summer and winter, respectively (Table 4). Sodium, among the cations ranged from 1.27 mg/l (spring) to 15.42 mg/l (summer), ammonium between 0.37mg/l (winter) to 63.16 mg/l (spring) and potassium between 0.57 mg/l (spring) to 7.05 mg/l (summer). Calcium showed the seasonal transition from a minimum of 3.55 mg/l (summer) to 17.50 mg/l (winter) while magnesium ranged between 1.19 mg/l to 3.69 mg/l during spring and winter, respectively (Table 5). Among the heavy metals, copper and nickel were recorded at below detection levels at few stations to a maximum of 67.0 μg/l (winter) and 88.0 μg/l (autumn), respectively. Zinc ranged between 2.0 μg/l (summer) to 60.0 μg/l (autumn) while cobalt and manganese showed the maximum value of 9.0 μg/l and 1700.0 μg/l recorded during the winter (Table 6).

**Table 1:** Information about different monitoring stations *viz.,* station number, name, code, geo-coordinates and altitude.

Station Number	Station Name	Station Code	Impact Category	Geo-Coordinates	Elevation (in m)
1	Thanalla I	ST-1a	Baseline Station	32°55′ 13.3″ N - 75⁰ 46′ 46.6″ E	2240
2	Thanalla II	ST-1b	Baseline Station	32°54′ 59.8″ N - 75° 46′ 13.5″ E	2184
3	Thanalla III	ST-1c	Baseline Station	32°54′ 59.9″ N - 75⁰ 45′ 43.4″ E	2156
4	Bheja I	ST-1d	Impact Station	32°56′ 28.5″ N - 75⁰ 45′ 10.5″ E	1823
5	Bhaja II	T-1	Impact Station	32°56′ 27.6″ N - 75⁰ 45′ 04.7″ E	1815
6	Thanthera	MC-1	Baseline Station	32°55′ 03.0″ N - 75⁰ 43′ 26.5″ E	2163
7	Puneja	T-3(I)	Impact Station	32°58′ 01.2″ N - 75⁰ 42′ 59.4″ E	1733
8	Dareja	MC-2	Impact Station	32°58′ 03.2″ N - 75⁰ 43′ 34.4″ E	1683
9	Gupt Ganga	MC-3	Impact Station	32°58′ 49.5″ N - 75⁰ 43′ 29.2″ E	1659
10	Atalgarh	T-2	Impact Station	32°59′ 06.6″ N - 75⁰ 43′ 50.1″ E	1636
11	Renda	MC-4	Impact Station	32°59′ 05.2″ N - 75⁰ 43′ 16.2″ E	1553
12	Dharampura	T-3(II)	Impact Station	32°58′ 49.4″ N - 75⁰ 42′ 57.4″ E	1638
13	Launcher Morh	ST-4a	Impact Station	32°58′ 39.7″ N - 75⁰ 42′ 07.1″ E	1682
14	Hallayan	ST-4b	Impact Station	32°58′ 27.5″ N - 75⁰ 42′ 22.6″ E	1700
15	College Road	T-4	Impact Station	32°59′ 15.1″ N - 75⁰ 42′ 44.0″ E	1572
16	Sarol Bagh I	T-3(III)	Impact Station	32°59′ 40.3″ N - 75⁰ 42′ 51.0″ E	1521
17	Sarol Bagh II	MC-5	Impact Station	32°59′ 45.3″ N - 75⁰ 42′ 43.2″ E	1510
18	Domail	MC-6	Impact Station	33^0^00′ 13.3″ N - 75⁰ 41′ 47.6″ E	1467
19	Hanga Nallah	T-5	Impact Station	33^0^00′ 30.8″ N - 75⁰ 41′ 37.9″ E	1480
20	Amira Nagar	MC-7	Impact Station	33^0^00′ 43.1″ N - 75⁰ 41′ 37.2″ E	1423
21	Drudu	MC-8	Impact Station	33^0^01′ 43.4″ N - 75⁰ 39′ 12.3″ E	1334
22	Mallothi	T-6(I)	Impact Station	33^0^02′ 46.9″ N - 75⁰ 36′ 42.3″ E	1357
23	Bhalla I	T-6(II)	Impact Station	33^0^04′ 02.3″ N - 75⁰ 36′ 45.1″ E	1202
24	Bhalla II	MC-9	Impact Station	33^0^04′ 12.9″ N - 75⁰ 36′ 43.3″ E	1185
25	Galgander	MC-10	Impact Station	33^0^08′ 07.5″ N - 75⁰ 33′ 44.4″ E	863

MC- Main Channel; T- Tributary; ST- Sub-tributary.

**Table 2:** Seasonal variation in physical parameters in Neeru stream.

Station	Winter				Spring				Summer				Autumn			
	Ta	Tw	Dis	Tur	Ta	Tw	Dis	Tur	Ta	Tw	Dis	Tur	Ta	Tw	Dis	Tur
MC-1 (S6)	4.1	3.0	20	0.1	9.6	6.4	139	2.1	17.3	12.4	65	1.13	12.6	10.4	27	0.1
MC-2 (S8)	6.3	4.1	56	2.0	13.4	9.4	291	12.1	21.3	15.9	145	0.85	14.8	11.7	79	0.2
MC-3 (S9)	6.8	4.7	59	1.8	13.1	9.8	294	4.2	20.3	15.2	151	1.45	14.8	11.5	83	0.2
MC-4 (S11)	8.0	6.0	81	1.3	15.5	11.2	327	5.6	22.3	17.7	168	3.75	16.3	12.4	104	1.3
MC-5 (S17)	8.0	5.6	96	1.7	14.3	11.1	403	5.7	22.1	17.7	206	2.80	15.6	12.4	127	1.4
MC-6 (S18)	8.0	6.2	111	2.2	16.0	12.6	490	8.8	23.3	19.1	237	2.32	16.3	12.9	141	1.3
MC-7 (S20)	8.6	6.5	120	1.7	15.1	12.0	531	7.0	22.8	18.5	264	4.50	17.5	14.0	161	0.3
MC-8 (S21)	8.0	5.9	124	0.9	15.5	11.2	536	5.3	23.6	18.8	263	2.58	17.3	14.0	166	3.2
MC-9 (S24)	10.0	7.9	180	0.8	17.0	11.9	725	4.1	25.1	19.9	392	5.97	19.0	14.6	228	1.1
MC-10 S25)	10.6	8.8	185	1.5	18.0	13.1	729	6.6	26.1	20.5	395	4.18	19.6	16.0	230	2.0
ST-1a (S1)	4.1	2.7	3	0	10.6	7.0	11	0	18.5	14.8	13	0	14.0	10.5	7	0.1
ST-1b (S2)	4.1	2.6	4	0	9.3	5.8	28	0	18.1	14.2	11	0	12.7	10.3	6	0
ST-1c (S3)	4.5	2.8	15	0	10.0	6.4	72	0	18.5	12.8	29	0	12.9	9.1	19	0
ST-1d (S4)	5.1	3.6	6	0.7	10.6	8.4	39	5.3	20.3	13.8	14	0	13.6	10.8	14	0.1
T-1 (S5)	5.1	3.6	29	0	10.9	8.2	153	1.9	20.0	13.3	72	7.62	13.6	10.9	46	4.6
T-2 (S10)	7.6	4.8	17	1.5	14.1	10.4	31	6.8	21.6	16.5	12	5.28	15.1	11.9	13	0.8
T-3(I) (S7)	6.5	5.4	5	3.2	13.1	10.9	73	0	21.1	18.0	19	0.18	14.8	12.1	14	1.1
T-3(II) S12)	6.3	4.4	6	61.4	12.6	9.1	58	12.6	19.8	15.0	19	22.03	14.0	10.9	16	33.3
T- 3 (III) (S16)	8.0	4.2	13	29.8	14.8	10.0	75	10.1	22.2	15.6	36	9.53	16.0	11.3	21	12.1
ST-4a (S13)	6.5	4.4	7	0.2	13.7	11.3	64	0.6	21.1	16.8	24	0	15.4	12.2	10	0.2
ST-4b (S14)	6.3	5.3	5	0.8	14.8	10.0	34	13.5	21.8	15.9	6	2.30	15.5	11.6	6	0.5
T-4 (S15)	7.3	5.9	17	42.0	14.6	11.7	113	13.6	21.5	18.5	37	5.17	15.1	12.7	21	15.9
T-5 (S19)	8.3	6.1	9	0.4	15.5	12.2	41	0.9	23.0	18.6	24	3.95	17.2	13.1	16	1.3
T-6(I) (S22)	7.3	5.7	45	0.4	14.1	10.0	148	0	23.0	18.1	91	1.25	16.3	12.7	43	1.5
T-6(II) S23)	9.6	7.5	58	0.4	15.0	12.1	185	0	24.3	19.6	128	4.03	17.3	13.5	60	3.1

Ta- Air temperature (°C); Tw- Water temperature (°C); Dis- Discharge (CFS); Tur-Turbidity (NTU).

**Table 3:** Seasonal variation in chemical parameters in Neeru stream.

Station	Winter					Spring					Summer					Autumn				
	pH	EC	TDS	DO	HCO_3_^–^	pH	EC	TDS	DO	HCO_3_^–^	pH	EC	TDS	DO	HCO_3_^–^	pH	EC	TDS	DO	HCO_3_^–^
MC-1 (S6)	7.4	57.1	37.1	11.5	28.8	7.5	39.6	25.8	10.0	13.9	7.8	33.6	30.1	9.1	15.5	7.6	48.1	31.1	9.8	25.5
MC-2 (S8)	7.4	98.8	53.5	11.3	33.6	7.6	57.3	37.1	9.8	17.3	7.8	62.5	40.6	8.9	22.4	7.5	88.6	57.5	9.8	30.3
MC-3 (S9)	7.4	112.7	72.5	11.2	33.8	7.5	63.8	41.4	9.8	17.1	7.8	62.8	40.5	8.9	19.4	7.5	96.8	63.0	9.5	29.9
MC-4 (S11)	7.4	120.6	78.5	11.2	34.3	7.5	66.1	43.0	9.9	16.7	7.8	69.5	45.0	8.7	22.6	7.6	105.0	68.1	9.4	33.7
MC-5 (S17)	7.4	133.4	86.5	11.1	38.3	7.6	70.1	45.8	9.9	16.3	7.8	79.1	51.3	8.7	21.7	7.5	117.1	75.8	9.4	33.3
MC-6 (S18)	7.4	142.0	91.9	11.1	36.7	7.5	84.6	54.7	9.9	20.5	7.8	88.1	57.3	8.6	22.2	7.5	122.6	79.8	9.7	38.1
MC-7 (S20)	7.4	139.0	90.1	11.2	38.6	7.4	72.6	47.1	9.8	18.3	7.6	87.3	56.5	8.4	22.1	7.5	124.1	80.1	9.3	34.4
MC-8 (S21)	7.3	150.3	97.8	11.1	32.8	7.5	78.0	50.6	9.7	16.4	7.8	92.6	60.5	8.5	19.8	7.6	132.6	86.1	9.5	31.0
MC-9 (S24)	7.4	133.2	94.5	11.3	39.5	7.6	71.8	46.6	9.6	18.8	7.8	95.3	61.8	8.5	22.0	7.6	132.3	85.6	9.5	34.8
MC-10 (S25)	7.4	135.8	99.5	11.1	39.8	7.6	73.4	47.6	9.7	19.5	7.8	101	65.5	8.3	21.1	7.6	137.3	89.0	9.3	32.9
ST-1a (S1)	7.4	63.9	41.5	11.4	23.3	7.6	48.3	31.1	10.0	13.3	7.8	58.1	40.6	8.8	16.4	7.6	64.6	42.3	9.7	22.6
ST-1b (S2)	7.4	50.5	32.9	11.6	30.3	7.5	35.3	22.8	10.1	16.0	7.8	38.5	25.0	8.8	14.0	7.6	47.1	30.6	9.7	25.6
ST-1c (S3)	7.4	70.5	45.6	11.5	23.7	7.5	41.6	26.8	9.8	12.0	7.8	33.8	22.1	8.8	10.8	7.6	57.8	38.0	9.8	19.3
ST-1d (S4)	7.4	173.8	113.0	11.3	31.1	7.6	118.3	76.6	9.8	17.4	7.8	166.3	107.8	8.9	20.6	7.6	167.5	137.6	9.6	28.4
T-1 (S5)	7.4	135.5	88.4	11.4	31.2	7.5	94.6	65.0	9.9	14.8	7.8	102.1	66.6	8.9	20.9	7.6	127.0	82.5	9.7	29.3
T-2 (S10)	7.4	131.4	87.6	10.9	32.2	7.5	107.8	70.0	9.6	17.1	7.7	137.8	89.8	8.5	21.9	7.5	122.3	80.8	9.6	30.7
T-3(I) (S7)	7.4	114.0	74.0	10.9	23.7	7.5	52.1	32.6	9.8	12.3	7.7	53.6	35.1	8.5	15.6	7.5	74.5	48.3	9.7	22.3
T-3(II) (S12)	7.4	159.7	103.5	10.0	27.0	7.7	64.1	41.6	9.2	12.8	7.8	97.1	63.0	7.7	17.2	7.5	129.1	83.8	9.1	24.3
T- 3 (III) (S16)	7.5	184.6	120.1	9.76	30.3	7.6	75.0	48.6	8.8	16.3	7.8	119.5	78.0	7.4	19.9	7.6	157.0	102.1	8.7	28.0
ST-4a (S13)	7.3	98.9	64.2	11.5	35.9	7.5	50.7	33.0	10.2	17.3	7.8	65.1	42.6	8.6	21.1	7.6	87.0	56.8	9.6	31.1
ST-4b (S14)	7.4	127.0	82.2	11.4	36.3	7.5	77.1	50.2	10.1	18.7	7.7	117.6	76.3	8.3	22.6	7.5	117.6	76.3	9.6	31.5
T-4 (S15)	7.4	155.2	101.4	10.2	39.4	7.5	69.5	45.2	9.5	16.0	7.8	118.0	77.0	8.4	20.1	7.5	144.5	94.3	9.5	32.0
T-5 (S19)	7.4	102.6	66.8	11.2	31.4	7.6	62.5	40.5	9.6	16.4	7.8	93.0	60.5	8.4	18.8	7.6	95.7	62.0	9.4	29.2
T-6(I) (S22)	7.4	81.5	52.6	11.4	25.7	7.6	43.6	27.0	9.9	12.5	7.9	57.6	37.3	8.8	15.4	7.6	64.5	41.8	9.5	23.9
T-6(II) (S23)	7.4	104.5	67.9	11.0	30.2	7.6	53.1	34.8	9.8	15.5	7.8	82.0	53.3	8.5	17.1	7.6	98.5	64.1	9.4	26.7

EC- Electrical Conductivity (μS/cm); TDS-Total Dissolved Solids (mg/l); DO-Dissolved oxygen(mg/l); HCO_3_^–^ - Bicarbonates(mg/l).

**Table 4:** Seasonal variation in major anions in Neeru stream.

Station	Winter					Spring					Summer					Autumn				
	F ^–^	Cl ^–^	NO_3_^–^	PO_4_^3 –^	SO_4_^2 –^	F ^–^	Cl ^–^	NO_3_^–^	PO_4_^3 –^	SO_4_^2 –^	F ^–^	Cl ^–^	NO_3_^–^	PO_4_^3 –^	SO_4_^2 –^	F ^–^	Cl ^–^	NO_3_^–^	PO_4_^3 –^	SO_4_^2 –^
MC-1 (S6)	< D.L	1.40	0.8	< D.L	3.07	0.06	0.79	3.10	0.02	1.30	0.07	0.62	2.07	0.17	1.86	0.07	1.01	1.11	< D.L	2.73
MC-2 (S8)	< D.L	2.12	0.77	< D.L	3.76	0.39	1.07	2.60	< D.L	1.33	0.24	1.11	2.10	< D.L	1.59	0.04	1.79	0.37	< D.L	3.88
MC-3 (S9)	0.02	2.72	1.37	0.04	2.18	0.23	1.47	3.76	0.12	1.46	0.14	1.75	2.19	0.11	3.44	0.04	2.27	0.72	0.09	2.79
MC-4 (S11)	0.05	3.21	0.45	0.03	4.30	0.11	1.69	3.85	0.12	2.0	0.03	1.59	2.06	0.10	3.08	0.02	2.42	0.82	0.06	3.50
MC-5 (S17)	< D.L	5.40	0.43	< D.L	2.74	0.11	1.74	2.93	0.06	1.81	0.11	2.58	1.09	0.01	3.0	0.03	4.57	0.68	< D.L	3.43
MC-6 (S18)	0.05	3.38	0.45	0.03	4.30	0.11	1.87	3.85	0.12	2.0	0.03	1.66	2.06	0.10	3.08	0.02	2.26	0.82	0.06	3.50
MC-7 (S20)	0.02	4.42	0.73	0.07	3.88	0.36	1.80	4.47	0.18	2.43	0.24	2.11	4.57	0.15	2.89	0.10	3.51	2.19	0.07	3.25
MC-8 (S21)	< D.L	4.66	0.28	< D.L	4.87	0.16	2.18	1.98	0.04	2.55	0.13	2.46	0.99	0.04	3.89	0.07	4.76	0.11	< D.L	5.12
MC-9 (S24)	< D.L	4.29	1.04	< D.L	3.25	0.12	2.17	5.98	0.08	2.16	0.12	2.48	3.26	0.09	3.0	0.06	4.17	1.29	0.06	3.28
MC-10(S25)	< D.L	5.01	0.65	< D.L	3.57	0.10	2.49	2.63	0.03	2.02	0.13	2.46	1.94	0.02	3.15	0.05	4.61	0.65	< D.L	3.60
ST-1a (S1)	< D.L	1.57	2.15	< D.L	2.50	0.05	0.82	2.97	< D.L	1.78	0.21	0.49	5.82	< D.L	3.53	0.10	1.31	1.70	< D.L	3.52
ST-1b (S2)	< D.L	0.68	1.75	< D.L	1.97	0.04	0.24	2.95	< D.L	1.16	0.04	0.28	2.48	< D.L	0.69	0.01	0.64	1.49	< D.L	2.06
ST-1c (S3)	< D.L	1.18	2.06	< D.L	2.29	0.06	0.52	3.29	< D.L	1.27	0.09	0.57	2.36	< D.L	1.26	0	0.77	1.41	< D.L	2.26
ST-1d (S4)	< D.L	2.38	0.97	< D.L	8.86	0.25	1.10	2.23	< D.L	5.97	0.07	0.92	2.39	< D.L	5.67	0.01	1.88	1.29	< D.L	8.01
T-1 (S5)	0.01	1.92	1.77	< D.L	7.35	0.14	1.06	3.67	< D.L	3.58	0.18	1.03	0.35	< D.L	3.61	0.09	1.53	0.62	< D.L	6.35
T-2 (S10)	0.01	3.49	1.17	0.04	3.86	0.28	2.13	3.05	0.23	3.03	0.15	2.37	2.42	0.16	3.55	0.05	2.88	0.48	0.07	3.44
T-3(I) (S7)	0.03	2.75	0.08	< D.L	2.81	0.35	1.58	1.90	< D.L	1.49	0.09	1.57	1.35	< D.L	1.17	0.10	2.36	0.35	< D.L	2.18
T-3(II) (S12)	0.02	9.33	7.07	0.06	2.20	0.38	3.8	4.28	0.18	1.37	0.14	3.41	3.33	0.14	2.46	0.07	6.49	5.58	0.09	2.49
T- 3 (III) (S16)	< D.L	9.08	6.54	0.08	2.09	0.12	4.22	3.35	0.12	1.67	0.09	4.91	4.07	0.08	2.35	0.01	9.45	5.91	0.07	2.16
ST-4a (S13)	0.05	1.30	0.74	< D.L	3.17	0.46	0.68	2.91	0.12	1.39	0.11	0.77	1.37	0.05	3.33	0.01	1.15	0.60	< D.L	3.0
ST-4b (S14)	0.05	2.60	0.52	0.04	2.39	0.36	1.44	1.88	0.19	1.64	0.12	2.13	1.57	0.12	2.65	0.11	2.71	0.38	0.06	2.75
T-4 (S15)	< D.L	7.48	7.04	0.14	1.91	0.20	3.82	3.90	0.21	1.59	0.2	3.94	4.53	0.20	1.79	0.06	7.11	5.89	0.13	2.06
T-5 (S19)	0.01	3.76	0.48	< D.L	2.05	0.14	1.68	2.52	0.07	1.31	0.15	1.84	1.05	0.02	2.07	0.04	2.84	0.18	< D.L	1.77
T-6(I) (S22)	< D.L	2.64	0.13	< D.L	1.59	0.13	1.35	2.34	0.02	1.09	0.16	1.67	2.20	0.05	1.38	0.08	2.36	0.22	< D.L	1.33
T-6(II) (S23)	0.03	2.92	0.77	< D.L	2.26	0.12	1.59	2.12	0.09	1.38	0.13	2.20	1.26	0.09	1.95	0.10	2.67	0.52	< D.L	2.75

F ^–^ - Flouride; Cl ^–^ - Chloride; NO_3_^–^ - Nitrate; PO_4_^3 –^ - Phosphate; SO_4_^2 –^ - Sulphate (all measured in mg/l).

**Table 5:** Seasonal variation in major cations in Neeru stream.

Station	Winter					Spring					Summer					Autumn				
	Na^+^	NH_4_^+^	K^+^	Ca^2+^	Mg^2+^	Na^+^	NH_4_^+^	K^+^	Ca^2+^	Mg^2+^	Na^+^	NH_4_^+^	K^+^	Ca^2+^	Mg^2+^	Na^+^	NH_4_^+^	K^+^	Ca^2+^	Mg^2+^
MC-1 (S6)	3.27	1.81	1.38	6.06	2.02	1.76	10.14	0.58	4.22	1.55	7.41	4.16	3.69	3.55	1.46	3.87	2.14	2.23	4.56	1.65
MC-2 (S8)	3.15	0.75	1.85	9.77	2.21	1.27	9.36	1.11	5.57	1.57	7.93	5.43	4.31	6.94	1.79	6.08	3.07	1.95	9.14	2.3
MC-3 (S9)	4.78	2.16	1.34	11.72	2.71	2.99	63.16	0.71	6.24	1.56	9.24	9.1	5.23	6.48	1.71	5.92	4.09	3.09	9.9	2.31
MC-4 (S11)	6.23	1.07	1.48	12.0	2.76	2.4	48.49	0.69	6.68	1.63	11.4	5.58	4.66	7.12	1.63	7.11	3.47	2.98	10.74	2.5
MC-5 (S17)	4.18	2.47	1.49	13.57	3.32	3.68	10.48	0.88	7.2	1.7	11.09	10.49	4.96	8.25	1.91	7.67	4.5	3.88	11.44	2.93
MC-6 (S18)	6.02	1.87	1.97	14.52	3.44	2.76	41.88	0.76	8.37	1.92	15.42	5.21	6.08	8.93	1.87	9.32	3.11	3.7	12.26	2.75
MC-7 (S20)	9.84	1.54	1.7	14.03	3.17	4.48	42.34	0.97	7.47	1.77	14.33	5.12	4.46	8.97	1.92	9.29	3.68	3.51	12.44	2.82
MC-8 (S21)	6.66	1.69	1.83	14.81	3.33	2.81	8.41	1.12	7.28	1.81	13.46	11.78	4.25	9.3	2.07	9.18	4.2	3.62	13.64	3.05
MC-9 (S24)	7.02	2.0	1.98	13.39	2.71	3.25	11.6	1.26	7.26	1.61	11.84	7.94	5.23	9.37	1.85	8.81	3.73	2.75	12.86	2.87
MC-10 (S25)	4.86	1.98	2.16	13.72	2.7	3.66	12.48	0.91	7.3	1.48	11.96	8.09	6.37	10.48	2.14	8.89	4.01	5.65	13.71	2.69
ST-1a (S1)	3.68	0.65	1.23	6.69	2.17	3.04	2.08	0.57	4.62	1.36	8.55	1.75	2.11	5.72	1.67	5.47	1.13	1.58	6.73	2.15
ST-1b (S2)	2.65	0.56	1.11	5.15	1.84	2.12	13.45	0.92	3.85	1.23	5.65	1.89	2.34	4.09	1.21	3.68	1.11	1.7	4.6	1.48
ST-1c (S3)	2.73	0.55	1.25	7.93	2.34	2.15	12.04	0.87	4.53	1.56	7.19	4.53	3.89	4.05	1.46	4.62	3.21	2.42	6.11	1.92
ST-1d (S4)	4.45	0.37	1.49	17.5	3.69	3.54	1.24	0.79	11.86	2.48	9.63	3.14	4.27	16.79	3.53	6.03	2.22	2.83	16.78	3.19
T-1 (S5)	3.74	0.85	1.29	13.58	2.9	2.45	13.38	0.81	9.53	1.82	8.84	5.11	4.51	10.37	2.08	5.27	2.98	2.58	12.55	2.58
T-2 (S10)	7.79	1.19	1.14	13.59	3.07	3.94	37.46	0.59	10.9	2.1	14.59	2.95	2.42	13.53	3.06	8.26	2.58	1.33	13.75	3.21
T-3(I) (S7)	7.71	0.56	1.43	11.75	2.79	2.38	12.18	0.83	5.47	1.8	10.14	4.58	5.56	5.62	1.95	6.73	2.75	3.01	7.45	2.4
T-3(II) (S12)	11.04	2.09	2.11	16.21	3.67	3.35	14.49	0.81	7.06	1.62	13.49	7.79	5.25	9.32	2.18	11	3.49	3.63	12.65	2.79
T- 3 III) (S16)	10.53	1.79	2.65	12.13	3.1	4.93	39.7	1.68	5.48	1.6	13.84	3.66	7.05	7.76	2	11.41	2.48	5.53	10.33	2.54
ST-4a (S13)	6.35	1.69	1.23	9.83	2.72	2.85	28.54	0.66	5.25	1.66	9.4	6.53	3.99	7.47	1.98	7.14	2.86	2.76	9.05	2.38
ST-4b (S14)	6.56	1.72	1.57	13.48	3.46	2.97	21.49	0.71	7.5	1.67	11.88	5.82	3.66	11.96	2.58	8.09	3.83	2.51	12.13	2.84
T-4 (S15)	10.82	1.97	1.91	15.72	3.57	3.96	52.86	1.01	7.91	1.72	13.35	6.56	3.94	11.8	2.56	10.32	3.09	3.35	14.33	3.16
T-5 (S19)	6.34	1.87	1.4	10.4	2.55	3.53	38.01	0.75	6.38	1.46	14.69	5.34	3.31	9.42	2.13	9.28	3.25	2.55	9.56	2.44
T-6(I) (S22)	5.22	1.75	1.46	8.22	2.36	2.6	12.65	0.91	4.36	1.31	13.58	6.19	2.84	5.87	1.58	9.34	3.61	1.9	6.3	1.65
T-6(II) (S23)	2.94	3.1	1.39	10.36	2.31	1.43	10.65	0.89	5.3	1.19	9.67	7.64	2.92	8.04	1.69	9.01	4.21	2.23	9.78	2.06

Na ^+^-Sodium; NH_4_^+^-Ammonium; K ^+^-Potassium; Ca ^2+^-Calcium; Mg ^2+^-Magnesium (all measured in mg/l).

**Table 6:** Seasonal variation in heavy metals in Neeru stream.

Station	Winter					Spring					Summer					Autumn				
	Cu	Ni	Zn	Co	Mn	Cu	Ni	Zn	Co	Mn	Cu	Ni	Zn	Co	Mn	Cu	Ni	Zn	Co	Mn
MC-1 (S6)	25.0	9.0	3.0	4.0	707.0	< D.L	4.0	5.0	1.0	43.0	< D.L	< D.L	3.0	< D.L	89.0	< D.L	33.0	2.0	< D.L	80.20
MC-2 (S8)	1.0	14.0	4.0	3.0	1086	< D.L	3.0	27.0	< D.L	< D.L	8.0	1.0	4.0	< D.L	< D.L	< D.L	3.0	3.0	1.0	59.0
MC-3 (S9)	4.0	17.0	6.0	4.0	1216	< D.L	4.0	12.0	4.0	385.0	1.0	3.0	9.0	1.0	13.0	2.0	3.0	13.0	1.0	1002
MC-4 (S11)	< D.L	13.0	18.0	2.0	882.0	1.0	2.0	7.0	< D.L	154.0	< D.L	1.0	8.0	2.0	54.0	< D.L	2.0	11.0	2.0	1272
MC-5 (S17)	2.0	1.0	6.0	2.0	1492	12.0	11.0	13.0	4.0	119.0	< D.L	< D.L	8.0	< D.L	276.0	6.0	2.0	6.0	< D.L	1229
MC-6 (S18)	2.0	13.0	23.0	7.0	1118	13.0	1.0	8.0	1.0	316.0	< D.L	< D.L	5.0	< D.L	134.0	2.0	< D.L	8.0	1.0	0985
MC-7 (S20)	2.0	5.0	10.0	6.0	1344	< D.L	2.0	7.0	< D.L	82.0	1.0	1.0	3.0	< D.L	235.0	< D.L	< D.L	5.0	1.0	1108
MC-8 (S21)	2.0	< D.L	9.0	2.0	1700	1.0	2.0	8.0	1.0	643.0	14.0	14.0	17.0	5.0	105.0	< D.L	2.0	6.0	< D.L	1377
MC-9 (S24)	< D.L	< D.L	7.0	1.0	1207	< D.L	< D.L	5.0	< D.L	196.0	2.0	3.0	4.0	< D.L	81.0	2.0	2.0	3.0	< D.L	1028
MC-10 (S25)	< D.L	< D.L	4.0	2.0	1269	< D.L	< D.L	4.0	< D.L	322.0	2.0	< D.L	4.0	< D.L	131.0	2.0	1.0	2.0	< D.L	80.2
ST-1a (S1)	1.0	9.0	31.0	7.0	287.0	12.0	1.0	17.0	3.0	31.0	4.0	2.0	14.0	2.0	< D.L	3.0	2.0	10.0	3.0	188.0
ST-1b (S2)	18.0	19.0	27.0	5.0	43.0	9.0	10.0	29.0	7.0	< D.L	3.0	5.0	14.0	1.0	< D.L	< D.L	1.0	7.0	1.0	459.0
ST-1c (S3)	3.0	6.0	11.0	6.0	629.0	1.0	6.0	12.0	< D.L	167.0	1.0	2.0	9.0	< D.L	154.0	2.0	< D.L	4.0	< D.L	296.0
ST-1d (S4)	2.0	2.0	5.0	1.0	88.0	< D.L	< D.L	5.0	< D.L	116.0	< D.L	< D.L	8.0	1.0	101.0	< D.L	2.0	6.0	2.0	827.0
T-1 (S5)	67.0	88.0	15.0	4.0	824.0	1.0	< D.L	5.0	1.0	73.0	4.0	1.0	5.0	< D.L	41.0	2.0	3.0	6.0	1.0	549.0
T-2 (S10)	< D.L	14.0	21.0	9.0	606.0	< D.L	5.0	6.0	< D.L	116.0	3.0	1.0	6.0	< D.L	145.0	< D.L	< D.L	8.0	1.0	575.0
T-3(I) (S7)	2.0	15.0	4.0	3.0	1133	< D.L	9.0	5.0	1.0	< D.L	< D.L	< D.L	5.0	< D.L	< D.L	< D.L	< D.L	7.0	1.0	813.0
T-3(II) (S12)	2.0	18.0	27.0	5.0	96.0	1.0	1.0	19.0	< D.L	262.0	1.0	< D.L	3.0	3.0	60.0	1.0	1.0	5.0	4.0	929.0
T- 3 (III) (S16)	1.0	< D.L	4.0	5.0	1275	< D.L	28.0	10.0	1.0	361.0	3.0	2.0	4.0	< D.L	15.0	2.0	2.0	6.0	4.0	1083
ST-4a (S13)	< D.L	15.0	6.0	2.0	1344	2.0	3.0	8.0	3.0	134.0	3.0	2.0	2.0	< D.L	116.0	< D.L	< D.L	2.0	< D.L	675.0
ST-4b (S14)	9.0	3.0	14.0	2.0	1479	7.0	< D.L	15.0	0	639.0	8.0	11.0	18.0	3.0	339.0	< D.L	3.0	4.0	1.0	1152
T-4 (S15)	5.0	8.0	6.0	2.0	1291	2.0	3.0	7.0	1.0	548.0	2.0	2.0	14.0	2.0	142.0	3.0	13.0	60.0	30.0	1181
T-5 (S19)	5.0	8.0	6.0	4.0	1303	< D.L	< D.L	7.0	1.0	232.0	1.0	1.0	3.0	< D.L	673.0	< D.L	< D.L	7.0	2.0	922.0
T-6(I) (S22)	1.0	2.0	7.0	1.0	1331	< D.L	14.0	6.0	< D.L	166.0	3.0	4.0	9.0	< D.L	401.0	< D.L	< D.L	5.0	< D.L	1258
T-6(II) (S23)	1.0	1.0	5.0	1.0	1509	< D.L	< D.L	5.0	< D.L	361.0	3.0	2.0	6.0	< D.L	115.0	3.0	1.0	5.0	< D.L	1047

Cu-Copper; Ni-Nickel; Zn-Zinc; Co-Cobalt; Mn- Manganese (all measured in μg/L).

Overall the fluctuations in physicochemical parameters indicated small scale changes associated with human activities along the course of the water body. A large set of parameters like air and water temperature, discharge, turbidity, electrical conductivity, TDS, HCO_3_, F, Cl, NO_3_^-^, PO_4_^3^, SO_4_^2^, Na^+^, NH_4_^+^, K^+^, Ca^2+^, Mg^2+^, Cu, Ni, Zn Co and Mn showed a rising trend down the stream with maximum values obtained for the autumn and winter seasons. The cations namely Na^+^, NH_4_^+^, K^+^, Ca^2+^, Mg^2+^ however, showed a negative trend along with dissolved oxygen as pH behaved neutral to slightly alkaline along the entire course. As typical of the hill streams, the river discharge and temperature broadly impact these concentrations.

**Fig. 2:**
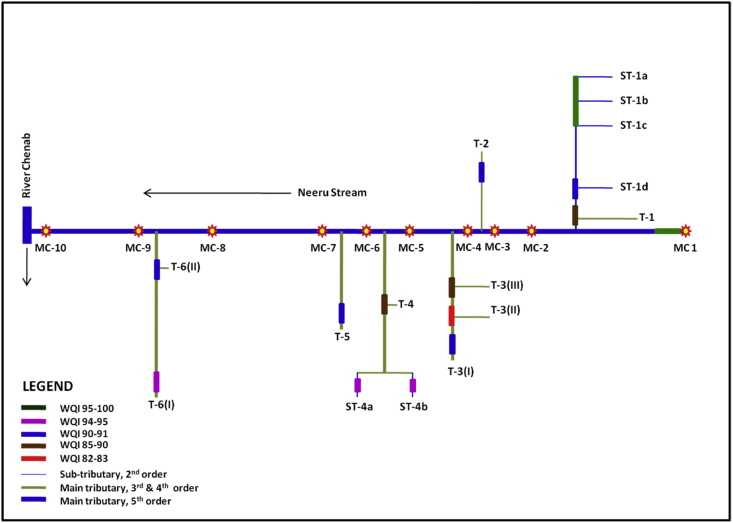
Schematic diagram indicating Water Quality Index in Neeru Stream.

We selected a set of 13 parameters (pH, turbidity, TDS, F, Cl, NO_3_^-^, SO_4_^2^, Ca^2+^, Mg^2+^, Cu, Ni, Zn and Mn) for generating the WQI, a mathematical framework calculated using ‘weighted arithmetic index’ (Table 7). The analysed results showed that all parameters were below the desirable limits except for turbidity (61.4 NTU) and nickel (88.0 μg/l) as drinking water quality standards [1]. The observed WQI values ranged from 82.97 to 95.50, thus indicating moderate to a high degree of pollution [2]. The headwaters MC-1, tributaries T-1, sub-tributaries ST-1a/1b/1c and ST-4a/4b indicated pristine water quality with WQI values observed between 94 and 95. The major tributaries T-2, T-3, T-5, T-6 (II) and sub-tributary ST-1d exhibited reasonably good water quality (WQI:90-91) while T-1, T-3(III) and T-4 flowing along the old Bhaderwah town revealed moderately high levels of pollution (WQI: 85-90). Tributary T-3 (II), which drains the city, emerged as the most polluted section with WQI of 82.97, the lowest for the whole Neeru catchment (Table 7, Fig. 2). Interestingly the main channel MC-2 to MC-10 revealed fairly good (WQI∼90) water quality (Fig. 2). The factors governing the deterioration of water quality include the pollutants from soluble salts (erosion and runoff), domestic (raw sewage, slaughterhouse and organic wastes) and agricultural activities. Most contaminated sections are the ones that pass through the urban sector. A steady rise in the water-borne infectious diseases in the past few years is attributed to the undesirable drinking water supplies in the region, the local health department claims. It thus becomes necessary to assess and monitor the stream water quality to examine its suitability for drinking and to adopt appropriate measures for the improvement of the drinking water supply system. The data presented in the paper are the part of investigations undertaken to determine the water quality characteristics of Neeru stream for land use characteristics and environmental factors.

**Table 7:** Canadian Water Quality Index for Neeru stream.

Station	WQI	Station	WQI
MC-1 (S6)	95.18	ST-1d (S4)	90.51
MC-2 (S8)	90.61	T-1 (S5)	86.21
MC-3 (S9)	90.19	T-2 (S10)	90.38
MC-4 (S11)	90.61	T-3(I) (S7)	90.74
MC-5 (S17)	90.14	T-3(II) (S12)	82.97
MC-6 (S18)	90.34	T- 3 (III) (S16)	87.89
MC-7 (S20)	90.01	ST-4a (S13)	94.64
MC-8 (S21)	90.00	ST-4b (S14)	94.03
MC-9 (S24)	90.48	T-4 (S15)	86.87
MC-10 (S25)	90.04	T-5 (S19)	90.43
ST-1a (S1)	95.50	T-6(I) (S22)	94.55
ST-1b (S2)	95.42	T-6(II) (S23)	90.23
ST-1c (S3)	95.32

## 2 Experimental design, materials and methods

### 2.1 Study area description

Neeru stream, a linear hydromorphological unit spanning 35 km in length originates at Kailash lake (3900 m) and drains into river Chenab near Pul-Doda at 850 m. 13 major tributaries contribute it during the entire course. Neeru watershed occupies rugged and mountainous terrain comprising of high ranges, deep valleys, cliffs and undulating meadows characterised with cold arid climate having short summers and long dry winters. The (average) temperature in the study area varies primarily with elevation as mercury dips to sub-zero in winters and reaches as high as 25 °C during summer while average annual precipitation received is 325 mm. The soils are mostly formed by the weathering of rocks, mainly the schist and granite. Forest, agriculture and built-up forms the major land use where agriculture and horticulture are the main drivers of the local economy. The tourism sector has started flourishing recently. Of the total population of 75,376 (39,051 males & 36,325 females), 64,292 people live in villages and suburbs [3]. Neeru stream, the lifeline of Bhaderwah offers a continuous and reliable source of fresh water for drinking (public water supply) and other purposes. The population influx, fast urbanisation and infrastructure expansion in the recent past have degraded the quality of this natural water body. The stream while passing through Bhaderwah town (38°58′48"N and 75°43′12"E, 1613 m) is exposed to a myriad of stressors, mostly anthropogenic.

### 2.2 Sample collection

We selected twenty-five sampling stations based on the size of the watershed, proximity to the habitation and area of influence. The pristine sections were taken as a baseline while the polluted sections as impact stations. Station names, codes and geo-coordinates are listed in Table 1 and presented in Fig. 1. We carried monthly sampling and analysis for two years (January 2014 to December 2015) and presented it on a seasonal basis.

### 2.3 Analytical procedure

The physicochemical parameters analysed included air temperature, water temperature, discharge, turbidity, pH, electrical conductivity, total dissolved solids, dissolved oxygen, bicarbonates, fluoride, chloride, sulphate, nitrate, phosphate, sodium, ammonium, potassium, calcium, magnesium, copper, nickel, zinc, cobalt and manganese. The air and water temperature, pH, electrical conductivity, turbidity, total dissolved solids (TDS), dissolved oxygen and salinity were recorded on the spot using bulb thermometer (for air temperature) and probe-based Horiba U-50 multi-parameter water quality analyser. We used the velocity-area method [4], [5] for calculating water discharge. The free carbon dioxide, carbonates and bicarbonates have been obtained by Titrimetry [7]. Three composite samples taken at 30 cm below the water surface were collected in the acid-washed 500 ml polyethene bottles from each location during the early mornings and transported to the laboratory for analysis [6], [7]. The cations (sodium, ammonium, potassium, calcium and magnesium), anions (fluoride, chloride, sulphate, nitrate and phosphate) and trace elements (copper, nickel, zinc, cobalt and manganese) were analysed using Ion Chromatograph (IC) 850-(Metrohm). We analysed the samples in aquatic ecology laboratory of Institute of Mountain Environment, Bhaderwah Campus and Ion Chromatography Laboratory of Department of Environmental Sciences, University of Jammu. The Canadian Water Quality Index [2] was calculated based on 13 parameters combined to produce a single value (between 0 and 100) based on scope, frequency and amplitude [8] that describes the water quality.
